# Comparative Analysis of the Nutritional Composition and Flavor Profile of Different Muscle Parts of Hybrid Abalone (*Haliotis discus hannai* ♀ × *H. fulgens* ♂)

**DOI:** 10.3390/foods14071265

**Published:** 2025-04-03

**Authors:** Tongtong Sun, Xiaoting Chen, Zhiyu Liu, Chenyang Xie, Shuji Liu, Yongchang Su, Nan Pan, Kun Qiao, Wenzheng Shi

**Affiliations:** 1 College of Food Science and Technology, Shanghai Ocean University, Shanghai 201306, China; m220300937@st.shou.edu.cn; 2 Key Laboratory of Cultivation and High-Value Utilization of Marine Organisms in Fujian Province, Fisheries Research Institute of Fujian, National Research and Development Center for Marine Fish Processing (Xiamen), Xiamen 361013, China; xtchen@jmu.edu.cn (X.C.); 13906008638@163.com (Z.L.); cute506636@163.com (S.L.); suyongchang@stu.hqu.edu.cn (Y.S.); npan01@qub.ac.uk (N.P.); 3 Engineering Technological Center of Mushroom Industry, Minnan Normal University, Zhangzhou 363000, China; m18652928716@163.com

**Keywords:** *Haliotis discus hannai* ♀ × *H. fulgens* ♂, muscle sites, nutritional evaluation, flavor profile

## Abstract

This study analyzed the basic nutritional components and amino acid, fatty acid, and mineral composition of hybrid abalone *Haliotis discus hannai* ♀ × *H. fulgens* ♂ adductor (AM), transition (TM), and skirt (SM) muscles. The taste characteristics of the muscles were measured via electronic tongue, and the volatile compounds were identified by headspace solid-phase microextraction-gas chromatography-mass spectrometry (HS-SPME-GC-MS) analysis. Compared to SM, AM and TM exhibited relatively similar basic nutritional compositions. Although SM exhibited the highest moisture content (84.67%), its protein content (only 11.83%) and total carbohydrate content (only 0.19%) were significantly lower than those of AM (20.42% and 4.14%) and TM (19.10% and 4.48%). The ash and fat contents were similar across the three muscle parts. The amino acid composition was consistent across three parts, and AM showed the highest total amino acid content, ratio of essential amino acids, and essential amino acid index. All three muscle parts were rich in polyunsaturated fatty acids, but the content was higher in AM and TM than in SM. The mineral elements were rich in variety, with high K, P, Mg, and Zn contents. Bitterness intensities were lower and umami and richness intensities were higher in AM and TM than in SM. The contents of volatile compounds related to fishy odor were higher in TM and SM than in AM. The results provided a scientific basis for the intensive processing and comprehensive utilization of *Haliotis discus hannai* ♀ × *H. fulgens* ♂.

## 1. Introduction

Abalone belongs to the phylum *Mollusca*, class *Gastropoda*, and family *Haliotidae* [1] and is an important marine economic aquaculture shellfish in China. Owing to its excellent taste, unique texture, and rich nutritional value, with high-protein and low-fat characteristics, it is highly appreciated by consumers worldwide [2]. In 2023, China’s abalone farming output was 245,000 tons, an increase of 7.26% compared to that of 2022, with Fujian Province leading production at 195,900 tons, accounting for approximately 80% of the national output [3]. The main economically important species of abalone distributed along the coast of China include *Haliotis discus hannai*, *Haliotis diversicolor*, *Haliotis asinine*, *Haliotis ovina*, *Haliotis planata*, *Haliotis varia*, and *Haliotis clathrata*, among others [4].

*Haliotis discus hannai* is a representative economic species of the northern *Haliotidae*, highly valued for its superb taste and favored by many consumers. As a northern temperate species, it has poor adaptability to the high temperatures of the southern summer. Despite the fact that *Haliotis discus hannai* has been acclimatized in the south for many years, being out of its natural habitat and frequent extreme weather events have led to low survival rates during the summer in Fujian coastal areas in recent years, with an increasing trend of high mortality rates, which has become one of the main issues hindering the development of abalone aquaculture in the province [5]. *H. fulgens* naturally inhabits the coastal regions of the United States and the western Pacific coast of Mexico and is a large, warm-water species with rapid development and high temperature tolerance, making it suitable for breeding fast-growing and heat-resistant varieties [6]. In 2018, Xiamen University bred a new variety of abalone, namely, *Haliotis discus hannai* ♀ × *H. fulgens* ♂ (variety registration number: GS-02-003-2018), using *Haliotis discus hannai* as the maternal parent and *H. fulgens* as the paternal parent. This new variety features rapid growth, high temperature tolerance, high summer survival rates, and the potential to grow large, and is gradually becoming the main variety promoted for abalone aquaculture in the Fujian Province [7].

The nutritional profiles, textural properties, and flavor characteristics of distinct muscle tissues in aquatic species exhibit significant heterogeneity [8,9,10]. These factors directly influence the processing suitability and food safety parameters of muscle tissues. Systematically investigating these variations can refine the manufacturing processes of aquatic products, enhance the quality of end-products, and support the development of value-added products that meet the evolving demands of the modern food market. The compositional components of muscles from different parts vary, especially in terms of protein distribution and hydration dynamics. This heterogeneity may pose technical challenges in maintaining product quality and preservation integrity during postharvest processing cycles [11]. For example, during the frozen storage period, the microbial diversity and textural properties of the abdomen and cheliped muscle of *Portunus trituberculatus* undergo significant changes. Accordingly, the cheliped muscle exhibits more severe quality deterioration than the abdomen muscle [12]. Moreover, owing to their varying protein compositions, muscles from different parts exhibit different changes in texture and digestibility during processing [13]. In abalone, the transition muscle has a higher collagen content and lower myofibrillar protein content than those of the adductor muscle, resulting in a lower degree of digestion after boiling [14]. Therefore, understanding the nutritional differences and quality characteristics of different muscle parts in aquatic products could help elucidate their nutritional value, enabling targeted processing and utilization.

At present, both domestic and international research on *Haliotis discus hannai* ♀ × *H. fulgens* ♂ mainly focuses on aquaculture, biology [15,16], and disease prevention and control [17]. There are also related studies on nutrition and flavor that primarily focus on overall nutritional evaluation and quality analysis. Zeng et al. [18] determined the nutritional components and textural characteristics of *Haliotis gigantea*, *Haliotis discus hannai*, *H. fulgens*, and their hybrid abalones *Haliotis discus hannai* ♀ × *H. fulgens* ♂ and *H. gigantea* ♀ × *Haliotis discus hannai* ♂. The findings showed that *Haliotis discus hannai* ♀ × *H. fulgens* ♂ has superior nutrition and flavor but inferior textural characteristics. Ma et al. [19] further studied the interactive effects of feed (*Amaranthemus tridentatus* and artificial feed) and water temperature on the nutritional value, flavor, and sensory qualities of *Haliotis discus hannai* ♀ × *H. fulgens* ♂ muscle. The results indicated that at low water temperatures, providing nutritionally balanced artificial feed can maximize abalone growth without affecting meat quality. Yu et al. [20] studied the changes in the nutritional components and textural characteristics of *Haliotis discus hannai* and *Haliotis discus hannai* ♀ × *H. fulgens* ♂ in different seasons, showing that the flavor, nutritional value, and taste of the abalones are best in winter. However, few studies have comprehensively evaluated the nutritional qualities of different parts of *Haliotis discus hannai* ♀ × *H. fulgens* ♂ muscle and determined flavor profiles and differences in volatile odor components. Therefore, the present study determined the basic nutritional components, including minerals, amino acids, and fatty acid composition, of the muscle of three parts of *Haliotis discus hannai* ♀ × *H. fulgens* ♂ abalone, namely, the adductor muscle (AM), transition muscle (TM), and skirt muscle (SM). Further, the nutritional and flavor characteristics of the different muscles were analyzed by using headspace solid-phase microextraction coupled to gas chromatography-mass spectrometry (HS-SPME-GC-MS) and an electronic tongue to provide a scientific basis for the intensive processing and comprehensive utilization of *Haliotis discus hannai* ♀ × *H. fulgens* ♂.

## 2. Materials and Methods

### 2.1. Materials and Reagents

Seventeen mixed amino acid standards, cysteine, and tryptophan were acquired from Sigma-Aldrich (St. Louis, MO, USA); 37 fatty acid methyl ester standards, nonadecanoic acid, and nonadecanoic methyl ester standards were acquired from Anpu Science and Technology (Shanghai, China); petroleum ether, anhydrous copper sulfate, potassium sulfate, boric acid, hydrochloric acid, concentrated sulfuric acid, trichloroacetic acid, NaOH (all analytical grade), n-hexane and boron trifluoride-methanol (chromatographic grade), and 2,4,6-trimethylpyridine (TMP, chromatographic grade) were acquired from Sinoreagent Chemical Reagent Co. (Shanghai, China).

### 2.2. Sample Preparation

*Haliotis discus hannai* ♀ × *H. fulgens* ♂ (average weight 215.50 ± 11.70 g per individual) was purchased from an abalone farm in Dongshan Island (Xiamen, China). The shells were removed, and the three muscle parts were finely dissected using a scalpel. Each part was then minced into small pieces with a kitchen knife. Finally, the muscle pieces from each part were thoroughly ground and homogenized using a mixer (JYL-D020, Joyoung, Shandong, China). Homogenized samples were then stored at −20 °C for further analysis.

### 2.3. Experimental Methods

#### 2.3.1. Determination of Proximate Composition

The approximate composition of abalone muscle was evaluated in accordance with the National Food Safety Standards of China. Moisture content was determined by drying 2 g of each muscle tissue part at 105 °C for 24 h and calculated based on the change in weight before and after drying. For ash content analysis, 5 g of each muscle tissue part was placed in a silicon crucible, carbonized on a heating plate, and subsequently heated in a muffle furnace at 550 °C for 4 h. The cooled samples were then reweighed to calculate the ash content. One gram of each muscle tissue part was weighed and digested in a digestive tube at 420 °C. Crude protein was measured using an automatic Kjeldahl nitrogen analyzer (Kjeltec 8400; FOSS A/S, Hillerød, Denmark) after digesting 1 g of abalone muscle at 420 °C. Fat content was evaluated via Soxhlet extraction (Soxtec 2050; Isenso, Shanghai, China). Homogenized samples were extracted with anhydrous ether for 6 h, dried at 100 °C for 1 h, and cooled for 0.5 h before calculating the crude lipid content. The polysaccharide content in abalone muscle was determined according to phenol-sulfuric acid spectrophotometry [21].

#### 2.3.2. Amino Acid Analysis

The amino acid composition was measured based on the method reported by Yu et al. [20], with some modifications. The samples were subjected to hydrolysis using hydrochloric acid, followed by analysis with an L-8800 automatic amino acid analyzer (Hitachi, Tokyo, Japan). Tryptophan in the samples was hydrolyzed with lithium hydroxide solution, and its content was measured using a fluorescence-based method.

#### 2.3.3. Fatty Acid Analysis

The extraction of total lipids from the samples and their conversion to methyl ester derivatives were performed according to the method reported by Yu et al. [20] with slight modifications. Samples from different muscle parts were hydrolyzed with hydrochloric acid. Subsequently, a chloroform-methanol solution (2:1, *v*/*v*) was added to the hydrolyzed samples for lipid extraction. The extracted lipids were mixed with 0.5 M NaOH-methanol and reacted in 80 °C water for 10 min until complete oil droplet dissolution. After cooling, a BF_3_-methanol solution (w = 15%) was added to the mixture and incubated in a water bath at 80 °C for 5 min. Subsequently, n-hexane and a saturated sodium chloride solution were added to the mixture. The solution was shaken vigorously to promote layer separation. The upper hexane layer was collected and filtered through a 0.22 μm organic phase membrane for subsequent GC-MS analysis. Fatty acids (FAs) were analyzed using a QP2020 NX gas chromatography-mass spectrometer (Shimadzu, Tokyo, Japan) fitted with an SH-Wax capillary column (30 m × 0.25 mm × 0.25 μm). The FAs were identified by comparing their mass spectra with the W8N08 database. The results are expressed as the relative percentage of each fatty acid in the total fatty acid profile.

#### 2.3.4. Determination of Trace Elements

A microwave digestion system was applied to digest 0.5 g of each muscle tissue part. The resulting digest was subsequently injected into an inductively coupled plasma mass spectrometer (Agilent 7900; Agilent Technologies, Santa Clara, CA, USA). This instrument was used to detect the signal response values of the target elements and the internal standard elements. The concentrations of the target elements in the digest were then determined based on a calibration curve.

#### 2.3.5. Evaluation of Amino Acid Nutritional Value

The amino acid score (AAS), chemical score (CS), and essential amino acid index (EAAI) were calculated based on the amino acid scoring criteria proposed by the Food and Agriculture Organization/World Health Organization (FAO/WHO) and compared with the amino acid profile of egg protein [22]. The corresponding calculations are presented as follows:

(1) $$\mathrm{AAS}=\frac{\mathrm{limiting}\;\mathrm{amino}\;\mathrm{acid}\;\mathrm{in}\;1\;g\;\mathrm{of}\;\mathrm{test}\;\mathrm{protein}/\left(\mathrm{mg}\right)}{\mathrm{same}\;\mathrm{amino}\;\mathrm{acid}\;\mathrm{in}\;1\;g\;\mathrm{of}\;\mathrm{reference}\;\mathrm{protein}/\left(\mathrm{mg}\right)}$$

(2) $$\mathrm{CS}=\frac{\mathrm{limiting}\;\mathrm{amino}\;\mathrm{acid}\;\mathrm{in}\;1\;g\;\mathrm{of}\;\mathrm{test}\;\mathrm{protein}/\left(\mathrm{mg}\right)}{\mathrm{same}\;\mathrm{amino}\;\mathrm{acid}\;\mathrm{in}\;1\;g\;\mathrm{of}\;\mathrm{egg}\;\mathrm{protein}/\left(\mathrm{mg}\right)}$$

(3) $$\mathrm{EAAI}=\sqrt[{n}]{{(\mathrm{Leu}}^{a}{/\mathrm{Leu}}^{b}{)\times (\mathrm{Val}}^{a}{/\mathrm{Val}}^{b})\times \ldots {\times (\mathrm{Lys}}^{a}{/\mathrm{Lys}}^{b}{)\times (\mathrm{His}}^{a}{/\mathrm{His}}^{b})}$$

Note: Leu^a^, Val^a^, and His^a^ represent the essential amino acid content of the sample; Leu^b^, Val^b^, and His^b^ are the standardized scores of essential amino acids.

#### 2.3.6. Electronic Tongue Analysis

Taste analysis was performed using an electronic tongue (TS-5000Z; Insent, Atsugi-shi, Japan). The system included two reference sensors and six test sensors: AAE (umami and richness), CT (saltiness), CA0 (acidity), C00 (bitterness and aftertaste-B), AE1 (bitterness and aftertaste-A), and GL1 (sweetness). The procedure was adapted from the method described by Liang et al. [23], with minor modifications. Minced abalone samples (70.0 ± 0.1 g) were weighed into a beaker, and 210 mL of deionized water was added. The mixture was magnetically stirred for 5 min, followed by heating in a boiling water bath for 15 min and additional magnetic stirring for 30 min. After cooling, the mixture was centrifuged at 8000 rpm for 10 min at room temperature, and the supernatant was filtered. Thirty milliliters of the filtrate were transferred to a sample cup for electronic tongue analysis, which was conducted at room temperature.

Prior to sample analysis, the sensors were cleaned in the cleaning solution for 90 s, rinsed with the reference solution for 120 s, and then immersed in the sample solution for 30 s to collect data at 1 s intervals. The sensor response values at the 30th second were used as the raw data for the electronic tongue analysis (at which point the sensors were stabilized). To ensure the reliability of the results, four parallel analyses were performed, and the last three replicates were used. Principal component analysis (PCA) was used to analyze taste differences among the three muscle parts.

#### 2.3.7. HS-SPME-GC-MS Analysis

Samples of 5 g were precisely weighed, and 5 mL of saturated NaCl solution was added. The mixture was homogenized and transferred to a 50 mL headspace vial. Extraction was performed at 60 °C for 40 min. Following extraction, the SPME fiber (DVB/CAR/PDMS, 50/30 μm; Sigma-Aldrich) was promptly inserted into the gas chromatography-mass spectrometry (GC-MS) injector (QP2020; Shimadzu). The fiber was thermally desorbed at 230 °C for 5 min within the thermal desorption unit. The SPME needle was then removed after the desorption process was completed.

Chromatographic conditions were as follows: SH-Wax capillary column (length × inner diameter × film thickness: 30 m × 0.25 mm × 0.25 μm) (Shimadzu); temperature program: the initial temperature was 40 °C, which was maintained for 3 min, then increased to 150 °C at a rate of 4 °C/min, and finally increased to 240 °C at a rate of 10 °C/min and held for 5 min. The injector was operated in splitless mode, with high-purity helium (99.999%) serving as the carrier gas at a flow rate of 0.8 mL/min.

Mass spectrometry parameters were as follows: the ionization energy was 70 eV, the ion source temperature was 230 °C, the quadrupole temperature was 150 °C, the detector voltage was 0.1 kV, the transfer line temperature was 280 °C, and the mass scan range was *m*/*z* 35–500 a.m.u.

Volatile compounds were tentatively identified using the NIST 20 mass spectral library. Qualitative analysis was conducted by comparing retention indices (RI) with those of a homologous series of n-alkanes (C_5_–C_30_) as references. The content of volatile compounds (ng/g) was semi-quantitatively determined using an internal standard, following the procedure reported by Song et al. [24]. Odor activity values (OAV) were employed to assess the contribution of each compound to the overall flavor profile of different parts of *Haliotis discus hannai* ♀ × *H. fulgens* ♂. Specifically, an OAV ≥ 1 suggested a significant contribution to the aroma profile [25]. The three formulas used are presented below:

(4) $$\mathrm{RI}=100\times n+100\times \frac{{\mathrm{Rt}-\mathrm{Rt}}_{n}}{{\mathrm{Rt}}_{n+1}{-\mathrm{Rt}}_{n}}$$

(5) $$C=\;\frac{A\times m}{A_{1}\times M}$$

(6) $$\mathrm{OAV}=\;\frac{C}{T}\;$$

where Rt denotes the retention time of unidentified compound(s), Rt_n_ and Rt_n+1_ denote the retention time of n-alkanes, A_1_ represents the peak area of the volatile compound, and A TMP is the peak area of TMP, m is the weight of TMP (μg), M is the weight (g) of samples, C is the relative concentration of the volatile compounds, and T represents the odor threshold values obtained from the literature [25].

### 2.4. Statistical Analysis

Each experimental group was analyzed in triplicate, and the results are expressed as the mean ± standard deviation. Data processing was conducted using SPSS software (version 27.0; IBM SPSS, Armonk, NY, USA). One-way analysis of variance (ANOVA) was employed to assess statistical significance, followed by Duncan’s multiple range test for post hoc comparisons. A significance level of *p* < 0.05 was adopted.

## 3. Results and Discussion

### 3.1. Composition of Abalone

**Figure 1:**
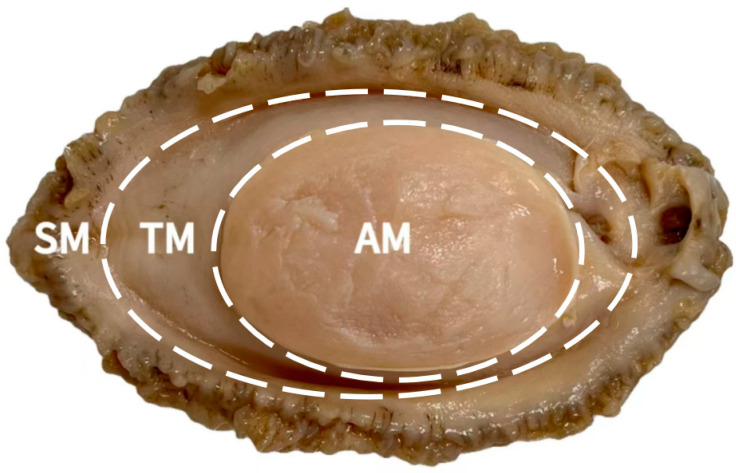
Division of *Haliotis discus hannai* ♀ × *H. fulgens* ♂ muscle. AM, adductor muscle; TM, transition muscle; SM, skirt muscle.

As shown in Figure 1, the abalone foot muscle was divided into three parts, namely, the AM, TM, and SM, for subsequent analysis.

Abalones are monovalve mollusks, and their entire muscle is the primary edible portion, unlike scallops, which are predominantly composed of adductor muscles. The main constituents of abalone muscle are myofibrillar proteins and collagen [26]. Table 1 presents the weight compositions of abalone foot muscle parts. As shown in the table, AM and TM accounted for a higher proportion of the foot (42.90% and 45.80%, respectively) than did SM. Thus, these two parts constitute a significant portion of abalone muscle, which is similar to the findings of Yin et al. [27]. However, the proportion of SM was relatively low (11.30%), which may be related to the species and size of the abalone. The entire foot muscle of *Haliotis discus hannai* ♀ × *H. fulgens* accounts for approximately 43% of the whole shell, which is higher than that of *Patinopecten yessoensis* (26.07%), *Ruditapes philippinarum* (around 20%), and *Argopecten irradians* (14.32%) [28,29,30].

### 3.2. Comparison of Nutritional Components in Different Muscle Parts of Haliotis discus hannai ♀ × H. fulgens ♂

#### 3.2.1. Composition Analysis

As illustrated in Table 2, significant differences were observed in the approximate compositions of the AM, TM, and SM of *Haliotis discus hannai* ♀ × *H. fulgens* ♂ (*p* < 0.05). Specifically, The SM moisture content was significantly higher than those of AM and TM (*p* < 0.05), whereas the total carbohydrate, ash, and crude protein contents of AM and TM were relatively similar and significantly higher than those of SM (*p* < 0.05). The fat content of TM was slightly higher than those of SM and AM. This is similar to the findings of Yin et al. [27]. The relatively low total carbohydrate content in SM may be attributed to its energy storage form. Generally, glycogen, the main form of energy storage in shellfish, varies in content with season and geographical origin [31]. Compared with that of other aquatic animals, the crude protein content of *Haliotis discus hannai* ♀ × *H. fulgens* ♂ is higher than that of *Mytilus galloprovincialis* (12.09%) [32], *Chlamys nobilis* (14.81%) [33], and *R. philippinarum* (6.91–10.39%) [30], while its crude fat content is much lower than that of *Corbicula fluminea* (1.59–6.50%) [34], *Mytilus edulis* (3.81%), and *Scapharca subcrenata* (2.70%) [35]. Consequently, *Haliotis discus hannai* ♀ × *H. fulgens* ♂ is a high-protein, low-fat aquatic product and can serve as a valuable source of animal protein for consumers.

#### 3.2.2. Amino Acid Composition and Content Analysis

The nutritional value of proteins is determined by the number, type, and proportion of amino acids they contain. Table 3 shows the amino acid composition and content in the AM, TM, and SM of *Haliotis. discus hannai* ♀ × *H. fulgens* ♂. All three parts contained 18 common amino acids required by the human body, comprising eight essential amino acids (EAAs), two semi-essential amino acids (semi-EAAs), and eight non-essential amino acids (NEAAs). The total amino acid content of AM, TM, and SM gradually decreased (*p* < 0.05). The EAA contents in AM and TM were higher than that in SM, with EAA/NEAA ratios of 66.92% and 55.49%, respectively, which were significantly higher than the ratio of 41.27% found in SM. The amino acid profiles of AM and TM were closer to the ideal protein model recommended by the FAO/WHO, indicating that these parts represent high-quality protein sources [36]. The amino acids whose content was increased across all three muscle parts (AM, TM, SM) were Asp, Glu, Gly, Leu, Lys, and Arg. Abalones are rich in lysine. Moderate consumption of abalones can help address the deficiency of lysine intake caused by a grain-based diet, thereby better supporting the growth and development of children. Leucine is capable of stimulating skeletal muscle growth and facilitating protein synthesis while also helping to regulate blood sugar levels. Supplementing with arginine in moderation can aid wound healing, with its mechanisms of action primarily involving the promotion of fibroblast proliferation and collagen synthesis [37].

#### 3.2.3. Evaluation of Amino Acid Nutritional Value

Foods rich in high-quality protein are of great value to humans. Protein quality is not only influenced by the content and types of amino acids but also by the proportion of essential amino acids (EAAs). The closer the EAA composition of food proteins is to that of the human body, the higher the quality of the protein. To further assess the nutritional value of the amino acids in the three parts of abalone muscle, EAAs were evaluated using the amino acid score (AAS) based on the FAO/WHO scoring model and the chemical score (CS) based on egg proteins. The results are summarized in Table 4. In both scoring models, Trp had the lowest score among all three muscle parts, making it the first limiting amino acid. The highest AAS scores for AM, TM, and SM were observed for Lys (1.19), Thr (0.96), and Thr (1.07), respectively. For CS, Thr had the highest score among the AM, TM, and SM, with values of 0.93, 0.82, and 0.91, respectively. The EAA index is used to characterize the similarity of the EAA content in food to that of standard proteins, and to some extent, it also reflects the digestibility and utilization of the protein [38]. The higher the essential amino acid index (EAAI), the better the protein quality. When the EAAI exceeds 0.95, it indicates that the protein is a high-quality source, with its EAA composition closely matching the requirements of the human body [39]. As shown in Table 4, the EAAI values of AM, TM, and SM decreased progressively, but all three were greater than 0.95, indicating that *Haliotis discus hannai* ♀ × *H. fulgens* ♂ muscle is a high-quality protein source. Overall, AM had the highest AAS, CS (except for Trp), and EAAI, indicating that it was the best protein source.

#### 3.2.4. Analysis of Fatty Acid Composition and Content

The quality of food fat is influenced not only by the composition and content of fatty acids but also by the proportional ratios of different fatty acid categories [40], with the latter being more critical. As shown in Table 5, the muscle tissues of different parts of *Haliotis discus hannai* ♀ × *H. fulgens* ♂ abalone exhibited minimal differences in fatty acid types, with a total of twenty fatty acids detected. Except for trace amounts of C18:3n6 in AM and TM, all other components were identical. These included nine saturated fatty acids (SFA), five monounsaturated fatty acids (MUFA), and six polyunsaturated fatty acids (PUFA). Additionally, the fatty acid composition pattern was consistent across all three parts in the following order: ∑SFA > ∑PUFA > ∑MUFA.

The SFA content ranged from 54.78 to 60.06%, with higher levels of palmitic and stearic acids. Palmitic acid can exert its role in preventing cardiovascular and cerebrovascular diseases through mechanisms such as modulating inflammatory responses and reducing the deposition of cholesterol in the arterial walls [41]. Stearic acid can regulate lipid metabolism, reduce the risk of atherosclerosis, promote mitochondrial biogenesis, and enhance energy utilization [42]. The unsaturated fatty acid (UFA) content was significantly higher in AM and TM than in SM (*p* < 0.05), with PUFA being the main component, accounting for approximately 30% of total fatty acids. These PUFAs consist of both *n*-3 and *n*-6 varieties, which are essential fatty acids for the human body and play a significant role in normal growth and development, cardiovascular health, and nervous system health [43]. All three parts contained relatively high levels of eicosapentaenoic acid (EPA), accounting for approximately 10.31–11.57% of total fatty acids, with SM having the lowest EPA content. Compared with other aquatic products such as *Pseudosciaena crocea* (EPA 4.45%, DHA 0.51%) [44], *Apostichopus japonicus* (EPA 6.93%, DHA 4.13%), and topaz sea cucumber (EPA 6.38%, DHA 5.01%) [45], the DHA content was lower, but the EPA content was higher, which is consistent with the findings of Yu et al. [20]. The ratios of ΣPUFA *n*-3/ΣPUFA *n*-6 in AM, TM, and SM were 0.52, 0.56, and 0.54, respectively, all of which were significantly higher than the FAO/WHO recommended values (0.1 and 0.2) for daily dietary [46]. Therefore, *Haliotis discus hannai* ♀ × *H. fulgens* ♂ could serve as an important dietary source of *n*-6 (C20:4 n6 and C18:2 n6) and *n*-3 (C20:5 n3 and C18:3 n3) fatty acids.

#### 3.2.5. Analysis of Mineral Element Content

**Figure 2:**
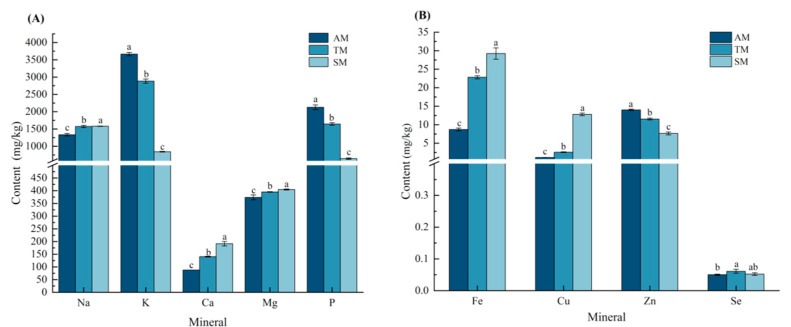
Mineral elements in different parts of *Haliotis discus hannai* ♀ × *H. fulgens* ♂ muscle (mg/kg). (**A**) Macroelements in different parts of *Haliotis discus hannai* ♀ × *H. fulgens* ♂ muscle. (**B**) Microelements in different parts of *Haliotis discus hannai* ♀ × *H. fulgens* ♂ muscle. Note: AM, adductor muscle; TM, transition muscle; SM, skirt muscle. Different superscript letters within the same element indicate significant differences (*p* < 0.05).

Minerals are essential nutrients that cannot be synthesized by the human body and must be obtained through diet [47]. Mineral elements include both macroelements and microelements; macroelements play a crucial role in human growth and development, whereas microelements are vital for important metabolic activities and biochemical processes. Ten mineral elements were measured, including five macroelements (K, Ca, Na, Mg, and P) and five microelements (Fe, Mn, Se, Cu, and Zn), as shown in Figure 2 (note that Mn was not detected).

All three parts of the *Haliotis discus hannai* ♀ × *H. fulgens* ♂ muscle were rich in a variety of mineral elements, but the content of Se was relatively low (the content in AM, TM, and SM is 0.050, 0.061, and 0.052 mg/kg, respectively), which is similar to the findings of Liu et al. [48]. Among the macronutrients, the Na and Mg contents were similar across the three parts, whereas the K and P contents in AM and TM were significantly higher than those in SM (*p* < 0.05). In contrast, SM had the highest Ca content. Among the microelements, the Zn content in AM was significantly higher than that in the other two parts (*p* < 0.05), whereas the Fe and Cu contents were the lowest. Phosphorus (P) is a component of nucleotides, ATP, and phospholipids, playing a crucial role in signal transduction and the coordination of cellular metabolism [49]. Magnesium has a wide range of functions in the human body, including maintaining the normal functions of nerves and muscles and acting as a cofactor for numerous enzymes involved in various biochemical reactions [50]. The K and Mg contents in *Haliotis discus hannai* ♀ × *H. fulgens* ♂ were similar to those in the muscles of salmon and tuna [51], suggesting it is a good dietary source of these minerals. Selenium, an essential trace element, functions as an integral component of selenoproteins to mediate critical metabolic processes. It plays a particularly important role in maintaining antioxidant defense systems and regulating thyroid hormone metabolism. Its deficiency is associated with compromised immune responses and an elevated risk of mood disorders [52]. Iron-deficiency anemia is caused by insufficient iron intake. Iron deficiency can affect the synthesis of hemoglobin, leading to a reduced capacity for oxygen transport, which in turn causes symptoms such as fatigue and somnolence [53]. Similar to magnesium, zinc functions as a cofactor for numerous enzymes and plays a crucial role in human growth and development as well as immune function [54]. The higher Zn and Fe contents in *Haliotis discus hannai* ♀ × *H. fulgens* ♂ were comparable to those of *Haliotis discus hannai* and *H. fulgens* [20]. In summary, *Haliotis discus hannai* ♀ × *H. fulgens* ♂ is rich in macroelements (such as K, Mg, and P) and microelements (such as Fe and Zn) and can serve as a good dietary source of minerals.

### 3.3. Analysis of Flavor Characteristics in Different Muscle Parts of Haliotis discus hannai ♀ × H. fulgens ♂

#### 3.3.1. Taste Analysis

**Figure 3:**
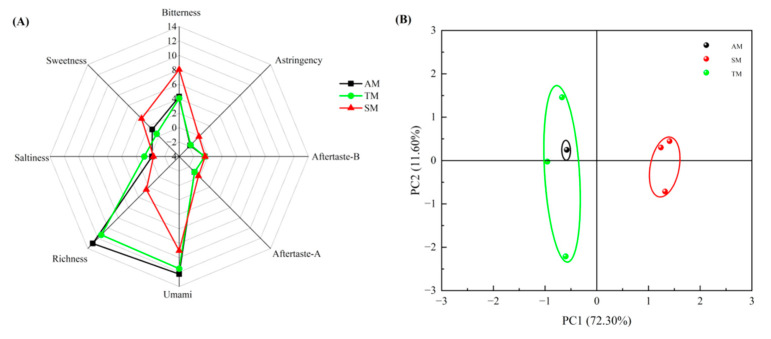
Electronic tongue analysis. (**A**) Radar chart of sensors of the different parts of *Haliotis discus hannai* ♀ *× H. fulgens* ♂. (**B**) PCA plot of the different parts of *Haliotis discus hannai* ♀ *× H. fulgens* ♂. AM, adductor muscle; TM, transition muscle; SM, skirt muscle; PCA, principal component analysis.

The electronic tongue is an analytical instrument that mimics human taste perception and is capable of conducting both qualitative and quantitative analyses of food taste [55]. The results showed that the sourness of all three parts of abalone muscle was below the tasteless point (−13), indicating that it is difficult to taste sourness in fresh abalone muscle. As shown in Figure 3A, there were differences in taste among the different parts of *Haliotis discus hannai* ♀ × *H. fulgens* ♂ muscle. The richness and umami response values of the three parts were significantly higher than other taste information, indicating that *Haliotis discus hannai* ♀ × *H. fulgens* ♂ muscle has an obvious umami characteristic. Compared to SM, AM and TM had lower bitterness intensity and higher umami and richness intensities, suggesting that the tastes of AM and TM are better, possibly owing to the amino acid compositions of the different parts [56].

As shown in Figure 3B, the first two principal components, PC1 (72.30%) and PC2 (11.60%), explained 83.90% of the total variance, which exceeds 80.00%, indicating that these two principal components can cover the vast majority of sample information. The PCA of the electronic tongue could distinguish the tastes of the three groups of samples. In the PCA plot of the electronic tongue (Figure 3B), AM and TM overlapped and were located on the same side of the coordinate axis, indicating that the tastes of the two parts were similar. In contrast, SM was distinctly separated from AM and TM and was located on the opposite side of the coordinate axis, indicating a significant difference in taste between the other two groups. The results showed that the tastes of AM and TM were similar, whereas that of SM was significantly different.

#### 3.3.2. Analysis of Volatile Compounds

##### Volatile Compounds and Contents

As shown in Table 6, 72 volatile compounds were detected in the three muscle parts of *Haliotis discus hannai* ♀ × *H. fulgens* ♂, including 21 aldehydes, five alcohols, 13 ketones, six hydrocarbons, three esters, eight acids, and 16 aromatic and other compounds. The three parts shared 32 common volatile compounds, 5 unique to AM, 5 unique to TM, and 12 unique to SM. Aldehydes, alcohols, ketones, hydrocarbons, and aromatic compounds constituted the main volatile substances in *Haliotis discus hannai* ♀ × *H. fulgens* ♂ muscle.

**Figure 4:**
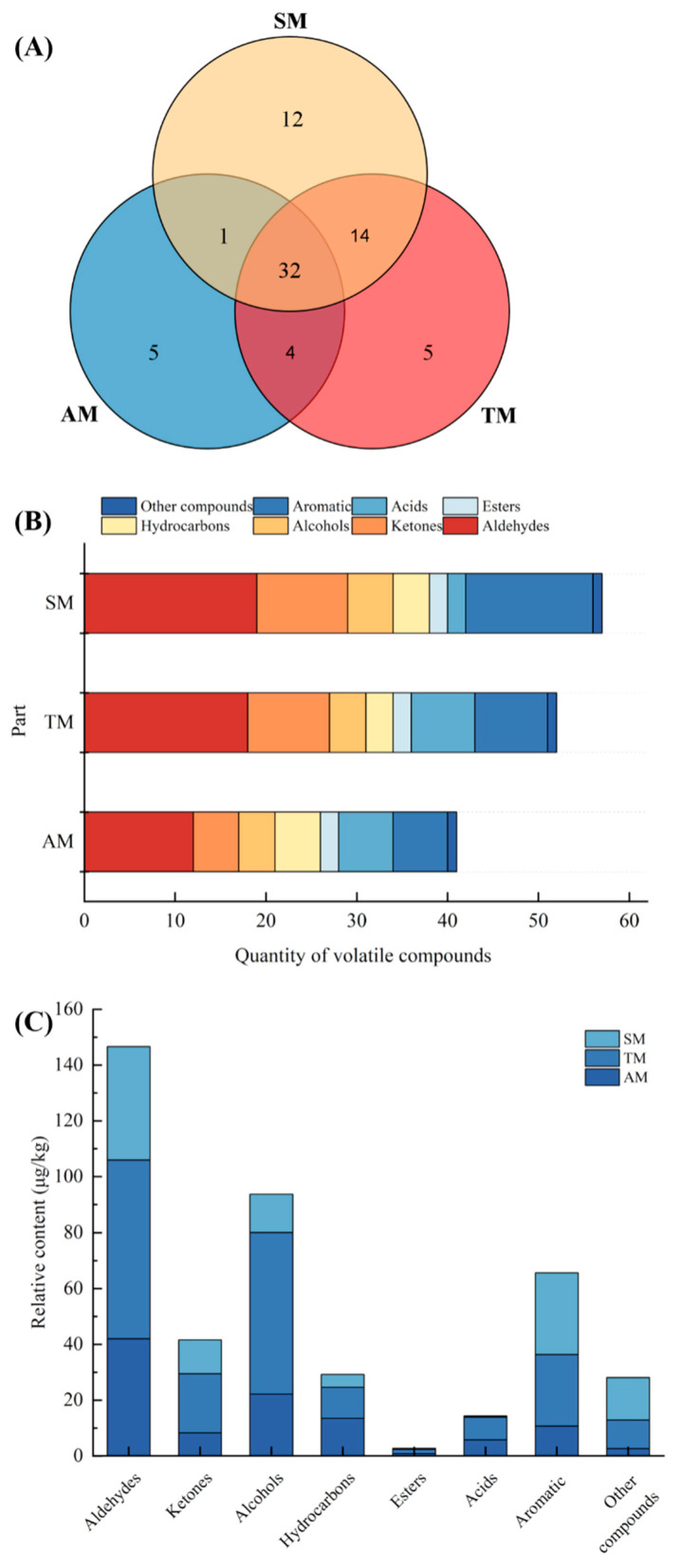
Volatile compounds in different parts of *Haliotis discus hannai* ♀ × *H. fulgens* ♂. (**A**) Venn diagram of volatile compound in different parts of *Haliotis discus hannai* ♀ × *H. fulgens* ♂. (**B**) Quantity of volatile compound classes in different parts of *Haliotis discus hannai* ♀ × *H. fulgens* ♂. (**C**) Relative content of volatile compound classes in different parts of *Haliotis discus hannai* ♀ × *H. fulgens* ♂. AM, adductor muscle; TM, transition muscle; SM, skirt muscle.

As shown in Figure 4, there were significant differences in the types and contents of volatile compounds among the three muscle parts. A total of 41, 54, and 58 volatile compounds were detected in AM, TM, and SM (Figure 4A), respectively. Further analysis of the relative contents of each class of compounds (Figure 4B) showed that the acid and ester contents were low in all three parts, whereas those of aldehydes, alcohols, ketones, and aromatic compounds were relatively high. TM had significantly higher aldehyde and alcohol contents than did AM and SM. SM had the highest content of aromatics and other compounds, whereas AM had the lowest content of all compound classes (except hydrocarbons). Overall, SM contained the highest number of volatile compounds, TM had the highest relative content of volatile compounds, and AM had the lowest (Figure 4C).

##### Clustered Heatmap and OPLS-DA

**Figure 5:**
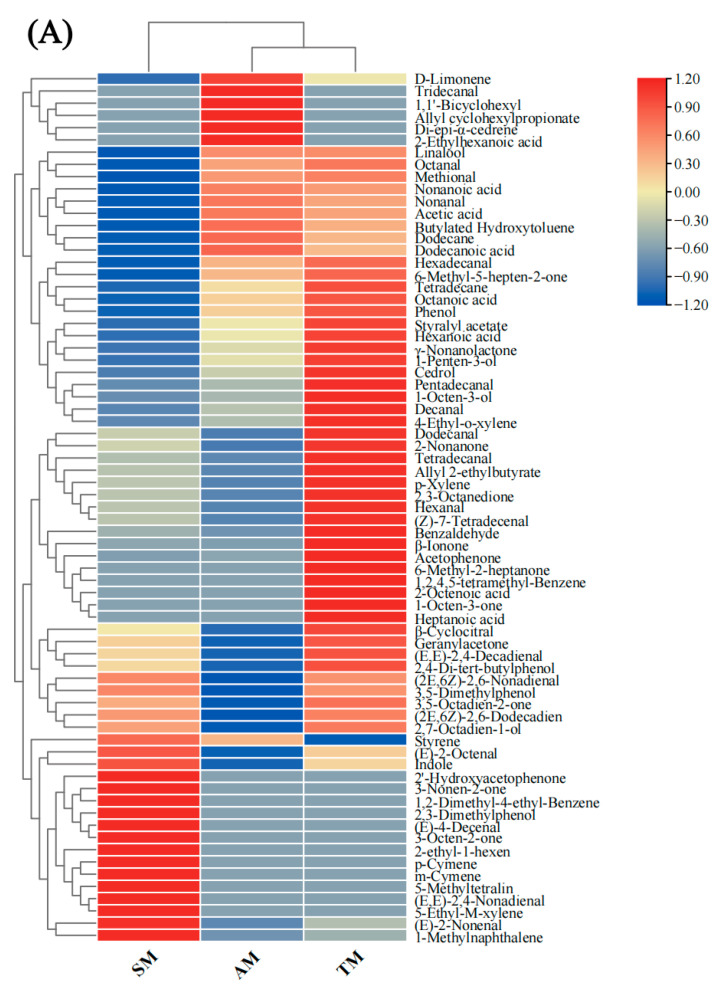

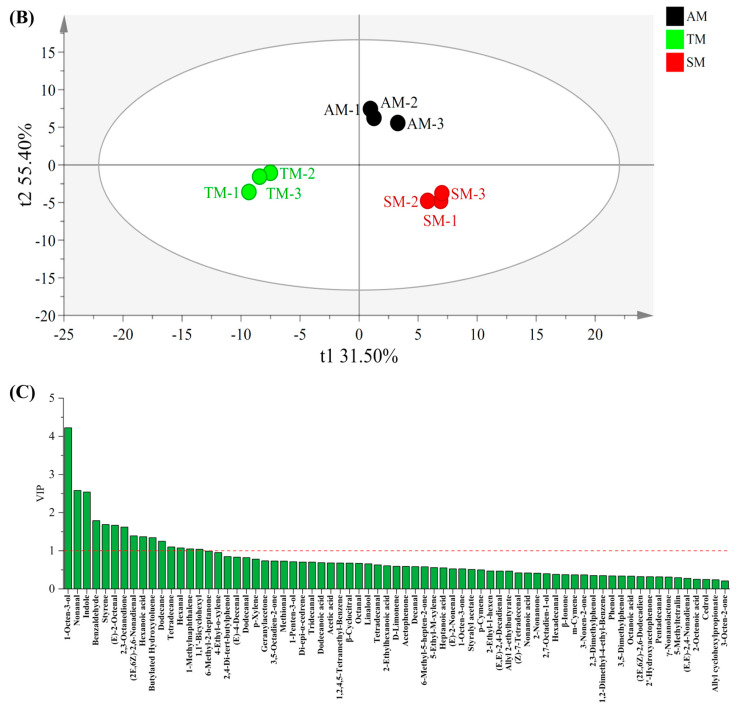
Comparison of volatile compounds in different parts of *Haliotis discus hannai* ♀ × *H. fulgens* ♂. (**A**) Heat map clustering of volatile compound in different parts of *Haliotis discus hannai* ♀ × *H. fulgens* ♂. (**B**) OPLS-DA score plot of volatile compound in different parts of *Haliotis discus hannai* ♀ × *H. fulgens* ♂. (**C**) VIP scores in OPLS-DA. AM, adductor muscle; TM, transition muscle; SM, skirt muscle; VIP, variable importance in projection.

The relative contents of the 72 volatile compounds identified by HS-SPME-GC-MS were normalized, followed by row standardization, and then a heatmap to further visualize the volatile compounds in the three parts. The heatmap (Figure 5A) shows that the volatile flavor compounds in the three muscle parts exhibit distinct color patterns, indicating differences in the composition and content of these compounds. The samples can be clustered into two groups based on the compound content: one group consists of AM and TM, and the other consists of SM. SM had higher contents of aromatic and other compounds, including indole, 1,2-dimethyl-4-ethyl-benzene, 2,3-dimethylphenol, p-cymene, m-cymene, 5-methyltetralin, 5-ethyl-m-xylene, and 1-methylnaphthalene, among others, whereas the other compound classes had relatively lower contents. AM and TM had similar volatile compounds, with relatively higher contents of aldehydes, alcohols, acids, and esters, such as linalool, cedrol, 1-penten-3-ol, methional, nonanal, acetic acid, and dodecanoic acid.

OPLS-DA was used to examine the correlations of key aroma compounds with different muscle parts. The parameters of the OPLS-DA model are shown in Figure 5B. The cumulative variance contribution value of the three samples was 87.00%, indicating significant differences in the volatile flavor compounds among the different parts. Variable importance in projection (VIP) was calculated to identify the volatile flavor compounds that contributed to the differences among the muscle parts. Generally, volatile flavor compounds with a VIP > 1 are key markers of sample flavor [57]. As shown in the VIP score plot (Figure 5C), 15 compounds with VIP > 1 were identified from all compounds, including five aldehydes (hexanal, nonanal, benzaldehyde, (E)-2-octenal, (2E,6Z)-2,6-nonadienal), one alcohol (1-octen-3-ol), one ketone (2,3-octanedione), three hydrocarbons (1,1′-bicyclohexyl, dodecane, tetradecane), one acid (hexanoic acid), and four aromatic and other compounds (styrene, 1-methylnaphthalene, butylated hydroxytoluene, indole). These volatile compounds could be used to distinguish the three samples.

##### Analysis of Key Volatile Compounds

**Figure 6:**
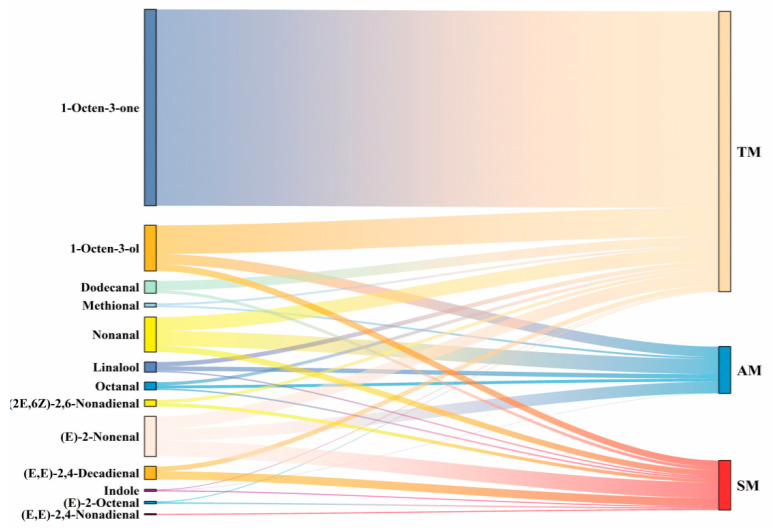
Sankey diagram of the OAVs of key active aroma compounds in different parts of *Haliotis discus hannai* ♀ × *H. fulgens* ♂. AM, adductor muscle; TM, transition muscle; SM, skirt muscle; OAV, odor activity value.

The odor activity value (OAV) can indicate the contribution of volatile compounds to the aroma of food. Typically, compounds with an OAV ≥ 1 are considered key flavor compounds, while those with an OAV between 0.1 and 1 play a role in modifying the overall flavor profile [58]. A total of 13 volatile compounds (OAV ≥ 1) were identified across the three parts, as shown in Figure 6. The key volatile flavor compounds in AM are octanal, nonanal, methional, (E)-2-nonenal, 1-octen-3-ol, and linalool. The key compounds in TM were octanal, nonanal, (E)-2-octenal, methional, (E)-2-nonenal, (2E,6Z)-2,6-nonadienal, dodecanal, (E,E)-2,4-decadienal, 1-octen-3-one, 1-octen-3-ol, and linalool. The key compounds in SM were octanal, nonanal, (E)-2-octenal, (E)-2-nonenal, (2E,6Z)-2,6-nonadienal, (E,E)-2,4-decadienal, dodecanal, (E,E)-2,4-nonadienal, 1-octen-3-ol, linalool, and indole.

Aldehydes, which are primarily produced by the oxidation of unsaturated fatty acids, have low thresholds and can significantly affect the flavor of aquatic products even at very low concentrations [59]. In total, 12, 18, and 19 aldehydes were detected in AM, TM, and SM, respectively, with the highest concentrations being hexanal, octanal, nonanal, and benzaldehyde. Hexanal has a grassy odor. Nonanal is one of the main contributors to the fishy odor, and benzaldehyde has a bitter almond-like aroma. Benzaldehyde is a common volatile compound in aquatic products, although its high threshold indicates that its flavor contribution is not significant. Alkenals, such as (E)-2-octenal, (E)-4-decenal, and (2E,6Z)-2,6-nonadienal, are associated with the formation of fishy odors.

Ketones are typically the degradation products of polyunsaturated fatty acids, amino acids, or microbial oxidation and are characterized by sweet, floral, and fruity odors [60]. 1-Octen-3-one and 6-methyl-2-heptanone were unique volatile compounds found in TM. 1-Octen-3-one has a very low threshold and can produce a strong, moldy, earthy odor, even at low concentrations. 6-Methyl-2-heptanone has an OAV between 0.1 and 1, indicating that it plays an important role in modifying the odor of abalone muscles.

Alcohols may originate from the oxidative degradation of PUFAs or the metabolism of carbonyl compounds. They are categorized into saturated and unsaturated alcohols, with the latter having lower sensory thresholds [61]. All three parts of the abalone muscle contained unsaturated alcohols, indicating their significant contribution to flavor. 1-Octen-3-ol and linalool are present in high amounts. 1-Octen-3-ol has a low threshold and primarily contributes to a fishy and mushroom-like odor that is commonly found in fish, shrimp, and shellfish. Linalool mainly has floral and fruity aromas, which give abalone muscle a pleasant odor.

Esters impart fruity, fatty, and floral odors to the aroma profiles of food. Their impact on the overall aroma of meat products is particularly notable, especially for short-chain esters that possess low detection thresholds [62]. Esters, including styralyl acetate, allyl 2-ethylbutyrate, and allyl cyclohexylpropionate, contributed to fruity, fatty, and floral aromas. In addition, a variety of acid compounds were detected in the three parts of the muscle, which is consistent with the results of Zheng et al. [63]. However, acids generally have high thresholds and little impact on odor. In addition to aldehydes, ketones, and alcohols, there were approximately 25% other compounds, mainly hydrocarbons and aromatic compounds. These compounds had low relative contents and high thresholds, and their contribution to odor was not significant (Table 6).

Hydrocarbons have a high threshold and usually contribute little to the flavor [64]. Sulfur-containing compounds are mainly derived from Strecker degradation of methionine and cysteine [65]. Sulfur-containing compounds have low thresholds and are important contributors to the characteristic aroma of fresh seafood. Methional was detected in AM and TM but not in SM, which may be related to the higher content of methionine and cysteine in AM and TM. A comprehensive analysis showed that TM had a stronger fishy odor, whereas AM had a better overall flavor.

This work provides some useful information for the study of the nutritional value and flavor characteristics of *Haliotis discus hannai* ♀ × *H. fulgens* ♂. However, there are some shortcomings. On the one hand, it lacks research on the taste substances in the muscle of *Haliotis discus hannai* ♀ × *H. fulgens* ♂ (such as free amino acids and taste nucleotides). On the other hand, it does not conduct an in-depth investigation into the formation mechanisms of volatile odor substances in different muscle parts.

## 4. Conclusions

*Haliotis. discus hannai* ♀ × *H. fulgens* ♂ muscle is nutritionally rich and beneficial, making it an ideal food that meets human health standards. However, the three parts of *Haliotis discus hannai* ♀ × *H. fulgens* ♂ muscle (AM, TM, and SM) showed significant differences in nutritional composition and flavor profile. Compared to SM, AM and TM had higher nutritional value, mainly reflected in protein content, amino acid, and fatty acid composition. In terms of flavor, AM and SM exhibited higher umami and richness intensity, while SM was characterized by a prominent bitter taste. Moreover, AM also had lower contents of 1-octen-3-one, 1-octen-3-ol, nonanal, and (E)-2-nonenal, which typically contributed to the fishy odor in raw materials. Systematically understanding the nutritional properties and flavor profiles of the different muscle parts of *Haliotis. discus hannai* ♀ × *H. fulgens* ♂ is of great significance for the quality control of its deep-processing. Future research and product development should focus on these characteristics, combining knowledge of nutrition and food science with culinary techniques to explore how to maximize the utilization of the properties of different parts of the hybrid abalone to meet the market’s demand for high-quality food.

## Figures and Tables

**Table 1:** The general weight compositions of *Haliotis discus hannai* ♀ × *H. fulgens* ♂ (%).

Position	AM	TM	SM
Ratio of abalone	42.90 ± 0.90 ^b^	45.80 ± 0.65 ^a^	11.30 ± 1.06 ^c^
Ratio of whole shell	18.42 ± 0.54 ^b^	19.67 ± 0.48 ^a^	4.85 ± 0.40 ^c^

Note: AM, adductor muscle; TM, transition muscle; SM, skirt muscle. Different superscript letters within the same row indicate significant differences (*p* < 0.05).

**Table 2:** Proximate composition in different parts of *Haliotis discus hannai* ♀ × *H.* fulgens ♂ (wet weight, w/%).

Position	Moisture	Protein	Fat	Ash	Carbohydrate
AM	73.08 ± 1.09 ^c^	20.42 ± 0.11 ^a^	0.69 ± 0.02 ^b^	1.34 ± 0.01 ^a^	4.14 ± 0.08 ^b^
TM	75.01 ± 0.12 ^b^	19.10 ± 0.12 ^b^	0.76 ± 0.03 ^a^	1.23 ± 0.01 ^b^	4.48 ± 0.08 ^a^
SM	84.67 ± 0.73 ^a^	11.83 ± 0.05 ^c^	0.66 ± 0.02 ^b^	0.83 ± 0.01 ^c^	0.19 ± 0.01 ^c^

Note: AM, adductor muscle; TM, transition muscle; SM, skirt muscle. Different superscript letters within the same column indicate significant differences (*p* < 0.05).

**Table 3:** Amino acid composition in different muscle parts of *Haliotis discus hannai* ♀ × *H. fulgens* ♂ (mg/g).

Amino Acid	AM	TM	SM
Ile	7.63 ± 0.14 ^a^	6.08 ± 0.20 ^b^	3.78 ± 0.14 ^c^
Leu	14.36 ± 0.19 ^a^	11.30 ± 0.40 ^b^	6.70 ± 0.20 ^c^
Lys	13.21 ± 0.19 ^a^	9.85 ± 0.52 ^b^	5.02 ± 0.20 ^c^
Met	4.14 ± 0.05 ^a^	3.40 ± 0.11 ^b^	1.94 ± 0.08 ^c^
Phe	5.75 ± 0.07 ^a^	4.90 ± 0.09 ^b^	3.48 ± 0.13 ^c^
Thr	8.84 ± 0.06 ^a^	7.32 ± 0.08 ^b^	5.04 ± 0.17 ^c^
Trp	1.26 ± 0.01 ^a^	1.11 ± 0.03 ^b^	0.82 ± 0.03 ^c^
Val	8.03 ± 0.11 ^a^	6.67 ± 0.16 ^b^	4.52 ± 0.16 ^c^
EAA	63.23 ± 0.79 ^a^	50.63 ± 1.51 ^b^	31.31 ± 1.08 ^c^
Arg	23.69 ± 0.21 ^a^	19.59 ± 0.12 ^b^	11.64 ± 0.31 ^c^
His	2.98 ± 0.05 ^a^	2.27 ± 0.08 ^b^	1.35 ± 0.06 ^c^
Semi-EAA	26.27 ± 0.25 ^a^	21.87 ± 0.18 ^b^	12.99 ± 0.37 ^c^
Ala	10.92 ± 0.06 ^a^	10.29 ± 0.12 ^b^	8.09 ± 0.22 ^c^
Asp	17.92 ± 0.16 ^a^	15.52 ± 0.21 ^b^	11.29 ± 0.31 ^c^
Glu	30.41 ± 0.28 ^a^	25.99 ± 0.40 ^b^	17.57 ± 0.47 ^c^
Gly	12.47 ± 0.17 ^b^	16.79 ± 1.63 ^a^	18.38 ± 0.52 ^a^
Pro	6.16 ± 0.04 ^c^	7.89 ± 0.67 ^b^	9.06 ± 0.22 ^a^
Ser	8.85 ± 0.04 ^a^	8.38 ± 0.14 ^b^	6.91 ± 0.19 ^c^
Tyr	5.95 ± 0.04 ^a^	4.82 ± 0.08 ^b^	3.21 ± 0.08 ^c^
Cys	1.79 ± 0.04 ^a^	1.58 ± 0.03 ^b^	1.35 ± 0.31 ^c^
NEAA	94.48 ± 0.28 ^a^	91.24 ± 1.92 ^b^	75.87 ± 1.92 ^c^
TAA	184.38 ± 1.31 ^a^	163.74 ± 0.63 ^b^	120.17 ± 3.29 ^c^
(EAA/TAA)/%	34.29	30.92	26.05
(EAA/NEAA)/%	66.92	55.49	41.27

Note: AM, adductor muscle; TM, transition muscle; SM, skirt muscle. Different superscript letters within the same row indicate significant differences (*p* < 0.05) and the same below.

**Table 4:** Comparison of AAS, CS, and EAAI in different parts of muscle in *Haliotis discus hannai* ♀ × *H. fulgens* ♂.

Amino Acid	Amino Acid Quality Score Based on FAO/WHO Scoring Model (mg/g)	Amino Acid Quality Score Based on Egg Protein (mg/g)	AM		TM		SM	
			AAS	CS	AAS	CS	AAS	CS
Thr	250	292	1.08	0.93	0.96	0.82	1.07	0.91
Val	310	410	0.79	0.60	0.70	0.53	0.77	0.58
Met + Cys	220	386	0.83	0.47	0.74	0.42	0.79	0.45
Ile	250	331	0.93	0.71	0.80	0.60	0.80	0.60
Leu	440	534	1.00	0.82	0.84	0.69	0.80	0.66
Phe + Tyr	380	565	0.94	0.63	0.84	0.56	0.93	0.63
Lys	340	441	1.19	0.92	0.95	0.73	0.78	0.60
Trp	60	99	0.64	0.39	0.61	0.37	0.72	0.44
EAAI			1.88		1.53		0.97	

Note: AAS, amino acid score; CS, chemical score; EAAI, essential amino acid index; AM, adductor muscle; TM, transition muscle; SM, skirt muscle.

**Table 5:** Fatty acid compositions and relative contents in different parts of *Haliotis discus hannai* ♀ × *H. fulgens* ♂ muscle (%).

Type	AM	TM	SM
C12:0	0.27 ± 0.02 ^a^	0.24 ± 0.01 ^a^	0.18 ± 0.01 ^b^
C13:0	0.15 ± 0.01 ^a^	0.15 ± 0.00 ^a^	0.15 ± 0.01 ^a^
C14:0	4.41 ± 0.09 ^c^	4.77 ± 0.12 ^b^	5.61 ± 0.15 ^a^
C15:0	2.97 ± 0.04 ^c^	3.14 ± 0.06 ^b^	3.84 ± 0.04 ^a^
C16:0	36.58 ± 0.05 ^b^	36.24 ± 0.11 ^c^	37.12 ± 0.06 ^a^
C17:0	2.30 ± 0.02 ^c^	2.38 ± 0.03 ^b^	2.62 ± 0.03 ^a^
C18:0	7.68 ± 0.02 ^c^	8.02 ± 0.01 ^b^	9.85 ± 0.16 ^a^
C20:0	0.11 ± 0.01 ^a^	0.09 ± 0.01 ^b^	0.10 ± 0.01 ^ab^
C24:0	0.30 ± 0.02 ^c^	0.42 ± 0.03 ^b^	0.58 ± 0.06 ^a^
∑SFA	54.78 ± 0.14 ^c^	55.44 ± 0.14 ^b^	60.06 ± 0.05 ^a^
C16:1-9c	1.94 ± 0.04 ^a^	1.80 ± 0.13 ^a^	0.94 ± 0.05 ^b^
C18:1t	8.62 ± 0.42 ^a^	7.92 ± 0.06 ^b^	7.50 ± 0.25 ^b^
C18:1 n9c	0.14 ± 0.00 ^a^	0.13 ± 0.00 ^b^	0.12 ± 0.01 ^b^
C20:1-11c	0.66 ± 0.03 ^b^	0.86 ± 0.08 ^a^	0.57 ± 0.01 ^b^
C22:1n9	0.21 ± 0.01 ^b^	0.23 ± 0.01 ^a^	0.20 ± 0.01 ^b^
∑MUFA	11.57 ± 0.47 ^a^	10.81 ± 0.21 ^b^	9.21 ± 0.22 ^c^
C18:2t	1.89 ± 0.17 ^a^	1.48 ± 0.05 ^b^	0.87 ± 0.08 ^c^
C18:3n6	0.11 ± 0.01 ^a^	0.10 ± 0.00 ^b^	ND
C18:3n3	0.42 ± 0.03 ^a^	0.46 ± 0.03 ^a^	0.45 ± 0.03 ^a^
C20:3n6	0.91 ± 0.02 ^a^	0.84 ± 0.05 ^a^	0.67 ± 0.05 ^b^
C20:4n6 (ARA)	19.19 ± 0.30 ^a^	19.17 ± 0.26 ^a^	18.32 ± 0.35 ^b^
C20:5n3 (EPA)	11.13 ± 0.20 ^b^	11.57 ± 0.14 ^a^	10.31 ± 0.07 ^c^
∑PUFA	33.66 ± 0.36 ^a^	33.62 ± 0.34 ^a^	30.61 ± 0.18 ^b^
∑UFA	45.23 ± 0.14 ^a^	44.43 ± 0.15 ^b^	39.82 ± 0.05 ^c^
∑PUFA *n*-3	11.55 ± 0.18 ^b^	12.02 ± 0.12 ^a^	10.76 ± 0.10 ^c^
∑PUFA *n*-6	22.10 ± 0.19 ^a^	21.60 ± 0.22 ^b^	19.85 ± 0.27 ^c^
n3/n6	0.52 ^b^	0.56 ^a^	0.54 ^a^

Note: AM, adductor muscle; TM, transition muscle; SM, skirt muscle. Different superscript letters within the same row indicate significant differences (*p* < 0.05). “ND” stands for not detected.

**Table 6:** Comparisons of the volatile compounds identified in different parts of *Haliotis discus hannai* ♀ × *H. fulgens* ♂ muscle by HS-SPME-GC-MS.

Volatile Compounds	Retention Time	LRI	Formula	CAS	Threshold (μg/kg)	Estimated Concentration (μg/kg)		
						AM	TM	SM
Aldehydes (21)						12	18	19
Hexanal	6.910	1049	C_6_H_12_O	66-25-1	5	1.18 ± 0.04	3.94 ± 1.39	1.91 ± 0.87
Octanal	13.903	1259	C_8_H16O	124-13-0	0.59	2.22 ± 0.37	2.39 ± 0.21	1.19 ± 0.55
Nonanal	17.587	1362	C_9_H_18_O	124-19-6	1.1	21.18 ± 4.03	19.30 ± 1.97	8.27 ± 2.09
(E)-2-Octenal	18.626	1392	C_8_H_14_O	2548-87-0	3	-	3.57 ± 0.15	5.60 ± 1.40
Methional	19.363	1413	C_4_H_8_OS	3268-49-3	0.45	1.07 ± 0.76	1.16 ± 0.10	-
Decanal	21.149	1465	C_10_H_2_0O	112-31-2	3	1.04 ± 0.23	1.89 ± 0.61	0.75 ± 0.06
Benzaldehyde	21.537	1477	C_7_H_6_O	100-52-7	750.89	10.26 ± 1.09	18.86 ± 5.59	11.39 ± 4.77
(E)-2-Nonenal	22.159	1495	C_9_H_16_O	18,829-56-6	0.19	2.82 ± 0.71	3.04 ± 0.49	3.79 ± 0.69
(E)-4-Decenal	22.443	1503	C_10_H_18_O	65,405-70-1	-	-	-	1.40 ± 0.39
(E,Z)-2,6-Nonadienal	23.758	1545	C_9_H_14_O	557-48-2	0.8	-	3.29 ± 0.02	3.42 ± 0.83
β-Cyclocitral	24.761	1577	C_10_H_16_O	432-25-7	3	-	1.06 ± 0.14	0.55 ± 0.09
(E,E)-2,4-Nonadienal	27.239	1654	C_9_H_14_O	5910-87-2	0.1	-	-	0.15 ± 0.00
Dodecanal	27.812	1673	C_12_H_24_O	112-54-9	0.13	-	1.54 ± 0.19	0.50 ± 0.12
Tridecanal	27.813	1672	C_13_H_26_O	10,486-19-8	10	0.86 ± 0.05	-	-
(Z)-7-Tetradecenal	28.869	1706	C_14_H_26_O	65,128-96-3	-	-	0.43 ± 0.07	0.11 ± 0.01
(E,E)-2,4-Decadienal	30.470	1762	C_10_H_16_O	25,152-84-5	0.027	-	0.47 ± 0.02	0.28 ± 0.05
2,6-Dodecadien-1-al	32.496	1846	C_12_H_2_0O	21,662-13-5	-	0.22 ± 0.00	0.42 ± 0.03	0.40 ± 0.12
γ-Nonanolactone	34.839	1977	C_9_H_16_O_2_	104-61-0	9.7	0.25 ± 0.14	0.41 ± 0.05	0.15 ± 0.01
Tetradecanal	35.104	1993	C_14_H_28_O	124-25-4	110	-	0.94 ± 0.13	0.21 ± 0.04
Pentadecanal	36.623	2096	C_15_H_30_O	2765-11-9	1000	0.47 ± 0.02	0.70 ± 0.05	0.41 ± 0.11
Hexadecanal	37.943	2195	C_16_H_32_O	629-80-1	-	0.44 ± 0.09	0.52 ± 0.01	0.18 ± 0.03
Ketones (13)						5	10	11
6-Methyl-2-heptanone	12.090	1208	C_8_H_16_O	928-68-7	8.1	-	2.11 ± 0.89	-
1-Octen-3-one	14.320	1271	C_8_H_14_O	4312-99-6	0.003	-	0.75 ± 0.42	-
2,3-Octanedione	15.252	1296	C_8_H_14_O_2_	585-25-1	-	3.54 ± 0.32	9.44 ± 2.31	5.09 ± 1.20
6-Methyl-5-hepten-2-one	15.575	1306	C_8_H_14_O	110-93-0	68	0.98 ± 0.31	1.21 ± 0.33	0.32 ± 0.07
2-Nonanone	17.384	1357	C_9_H_18_O	821-55-6	41	-	0.40 ± 0.27	0.14 ± 0.01
3-Octen-2-one	17.879	1370	C_8_H_14_O	1669-44-9	-	-	-	0.09 ± 0.03
3,5-Octadien-2-one	23.186	1527	C_8_H_12_O	38,284-27-4	-	-	0.09 ± 0.03	0.82 ± 0.11
Acetophenone	25.575	1603	C8H8O	98-86-2	65	1.37 ± 0.65	2.47 ± 0.78	1.34 ± 0.33
2′-Hydroxyacetophenone	29.982	1745	C_8_H_8_O_2_	118-93-4	-	-	-	0.21 ± 0.06
Geranylacetone	31.774	1811	C_13_H_22_O	3796-70-1	60	-	1.26 ± 0.27	0.80 ± 0.15
β-Ionone	33.420	1892	C_13_H_2_0O	14,901-07-6	-	0.78 ± 0.11	1.14 ± 0.11	0.79 ± 0.20
3-Nonen-2-one	35.560	2023	C_15_H_24_O_2_	14,309-57-0	800	-	-	0.27 ± 0.07
2,6-Di-tert-butyl-4-hydroxy-4-methylcyclohexa-2,5-dien-1-one	36.063	2058	C_15_H_24_O_2_	10,396-80-2	-	1.63 ± 0.43	2.29 ± 0.07	2.25 ± 0.20
Alcohols (5)						4	5	5
1-Penten-3-ol	9.509	1133	C_5_H_10_O	616-25-1	358.1	0.78 ± 0.21	1.52 ± 0.22	0.23 ± 0.05
1-Octen-3-ol	19.594	1419	C_8_H_16_O	3391-86-4	1.5	19.87 ± 3.21	54.4 ± 8.51	12.51 ± 0.61
Linalool	22.776	1514	C_10_H_18_O	78-70-6	0.22	1.24 ± 0.05	1.24 ± 0.11	0.38 ± 0.09
2,7-Octadien-1-ol	33.825	1914	C_8_H_14_O	23,578-51-0	-	-	0.30 ± 0.01	0.26 ± 0.06
Cedrol	36.343	2077	C_15_H_26_O	77-53-2	-	0.29 ± 0.16	0.44 ± 0.1	0.22 ± 0.03
Hydrocarbons (6)						5	3	4
D-Limonene	10.730	1169	C_10_H_16_	5989-27-5	34	0.98 ± 0.11	0.61 ± 0.04	0.28 ± 0.02
Dodecane	11.173	1182	C_12_H_26_	112-40-3	10,000	4.47 ± 1.13	3.66 ± 0.68	1.22 ± 0.38
2-Ethyl-1-hexene	14.334	1271	C_8_H_16_	1632-16-2	-	-	-	0.45 ± 0.12
Tetradecane	18.159	1280	C_14_H_30_	629-59-4	1000	5.33 ± 1.70	6.87 ± 0.77	2.64 ± 0.64
1,1′-Bicyclohexyl	18.620	1391	C_12_H_22_	92-51-3	-	1.86 ± 0.42	-	-
Di-epi-.α-cedrene	23.292	1530	C_15_H_24_	50,894-66-1	-	0.85 ± 0.06	-	-
Esters (3)						2	2	2
Styralyl acetate	27.373	1659	C_10_H_12_O_2_	93-92-5	160	0.66 ± 0.01	1.04 ± 0.26	0.30 ± 0.03
Allyl 2-ethyl butyrate	27.470	1662	C_9_H_16_O_2_	7493-69-8	-	-	0.50 ± 0.07	0.12 ± 0.02
Allyl cyclohexylpropionate	30.308	1756	C_12_H_2_0O_2_	2705-87-5	-	0.18 ± 0.00	-	-
Acids (8)						6	7	2
Acetic acid	19.154	1407	C_2_H_4_O_2_	64-19-7	99,000	1.11 ± 0.16	0.98 ± 0.07	0.20 ± 0.05
Hexanoic acid	31.341	1792	C_6_H_12_O_2_	142-62-1	890	2.22 ± 0.50	4.72 ± 0.42	-
2-Ethyl-hexanoic acid	33.596	1900	C_8_H_16_O_2_	149-57-5	27,000	0.63 ± 0.09	-	-
Heptanoic acid	33.597	1900	C_7_H_14_O_2_	111-14-8	640	-	0.66 ± 0.06	-
Octanoic acid	35.369	2011	C_8_H_16_O_2_	124-07-2	-	0.17 ± 0.04	0.27 ± 0.06	-
Nonanoic acid	36.828	2111	C_9_H_18_O_2_	112-05-0	-	0.57 ± 0.03	0.54 ± 0.05	0.20 ± 0.05
2-Octenoic acid	37.005	2125	C_8_H_14_O_2_	1470-50-4	-	-	0.17 ± 0.10	-
Dodecanoic acid	40.377	2413	C_12_H_24_O_2_	143-07-7	-	1.09 ± 0.58	0.79 ± 0.22	-
Aromatic compounds (15)						6	8	14
p-Xylene	8.658	1108	C_8_H_10_	106-42-3	1000	0.68 ± 0.12	0.68 ± 0.12	1.10 ± 0.29
Styrene	12.678	1225	C_8_H_8_	100-42-5	65	5.34 ± 1.13	-	7.12 ± 0.89
1,2,3-Trimethyl-benzene	13.504	1248	C_9_H_12_	526-73-8	-	2.33 ± 0.44	4.33 ± 0.74	1.76 ± 0.76
4-Ethyl-1,2-dimethyl-benzene	16.065	1319	C_10_H_14_	934-80-5	-	-	-	0.27 ± 0.16
1,2,4,5-Tetramethyl-benzene	18.464	1387	C_10_H_14_	95-93-2	-	-	1.02 ± 0.19	-
1-Ethyl-3,5-dimethyl-benzene	18.476	1387	C_10_H_14_	934-74-7	-	-	-	0.70 ± 0.47
p-Cymene	18.829	1397	C_10_H_14_	99-87-6	5.01	-	-	0.51 ± 0.14
m-Cymene	20.531	1447	C_10_H_14_	535-77-3	800	-	-	0.27 ± 0.04
1,2,3,4-Tetrahydro-5-methyl-naphthalene	26.939	1645	C_11_H_14_	2809-64-5	-	-	-	0.20 ± 0.11
1-Methyl-naphthalene	32.211	1832	C_11_H_10_	90-12-0	3	0.35 ± 0.08	0.65 ± 0.11	2.70 ± 0.80
2,3-Dimethyl-phenol	32.758	1859	C_8_H_10_O	526-75-0	500	-	-	0.28 ± 0.18
Butylated hydroxytoluene	33.059	1874	C_15_H_24_O	128-37-0	1000	-	16.6 ± 1.84	12.91 ± 3.74
Phenol	34.459	1953	C_6_H_6_O	108-95-2	58,585.25	0.54 ± 0.04	0.65 ± 0.04	0.36 ± 0.08
3,5-Dimethyl-phenol	36.931	2119	C_8_H_10_O	108-68-9	500	-	0.21 ± 0.05	0.22 ± 0.05
2,4-Di-tert-butylphenol	38.605	2250	C_14_H_22_O	96-76-4	500	1.46 ± 0.24	1.51 ± 0.20	0.88 ± 0.09
Other compounds (1)						1	1	1
Indole	40.021	2381	C_8_H_7_N	120-72-9	11	2.63 ± 0.34	10.24 ± 1.15	15.24 ± 0.65

Note: “-” indicates not detected. AM, adductor muscle; TM, transition muscle; SM, skirt muscle.

## Data Availability

The original contributions presented in this study are included in the article. Further inquiries can be directed to the corresponding authors.

## Abbreviations

The following abbreviations are used in this manuscript:

AAS	Amino acid score
AM	Adductor muscle
CS	Chemical score
EAA	Essential amino acid
EAAI	Essential amino acid index
EPA	Eicosapentaenoic acid
HS-SPME-GC-MS	Headspace solid-phase microextraction coupled to gas chromatography–mass spectrometry
MUFA	Monounsaturated fatty acid
NEAA	Non-essential amino acid
OAV	Odor activity value
PUFA	Polyunsaturated fatty acid
Semi-EAA	Semi-essential amino acid
SFA	Saturated fatty acid
SM	Skirt muscle
TM	Transition muscle
UFA	Unsaturated fatty acid
VIP	Variable importance in projection
